# When the Going Gets Challenging—Motivational Theories as a Driver for Workplace Health Promotion, Employees Well-Being and Quality of Life

**DOI:** 10.3390/bs13110898

**Published:** 2023-10-30

**Authors:** Lisa Karolin Coco, Petra Heidler, Holger Adam Fischer, Valeria Albanese, Roy Rillera Marzo, Vlastimil Kozon

**Affiliations:** 1 Equine Hospital “Tierärztliches Kompetenzzentrum für Pferde Grosswallstadt”, 63868 Grosswalstadt, Germany; 2 Department of Public Health, St. Elizabeth University of Health and Social Work, 811 02 Bratislava, Slovakia; 3 Institute International Trade and Sustainable Economy, IMC University of Applied Sciences, 3500 Krems, Austria; 4 Department of Health Sciences, St. Pölten University of Applied Sciences, 3500 Krems, Austria; 5 International Medical School, Management and Science University, Shah Alam 40100, Malaysia; 6 Global Public Health, Jeffrey Cheah School of Medicine and Health Sciences, Monash University Malaysia, Subang Jaya 47500, Malaysia; 7 Austrian Society for Vascular Nursing, 1090 Vienna, Austria; 8 Wound Diagnosis and Wound Management, 1090 Vienna, Austria

**Keywords:** well-being, quality of live, veterinary, healthcare, motivation, motivational theories, reward

## Abstract

Being characterized by high numbers of physical and mental health issues, the veterinary sector faces some negative peculiarities compared to other professions. To evaluate and possibly improve employees’ well-being and to reward according to individual motivation, managing staff and the profession in general must understand every individual’s motivation. Defining the motivation of veterinary professionals in a multidisciplinary way can be the first step in increasing the well-being of employees. To assemble current theories of work motivation and adapt and extend them to serve the veterinary profession, basic knowledge of the classical general psychological motivational theories and work motivational theories is inevitable. The authors aimed to illustrate the different motivational theories’ key aspects and historical evolution to provide veterinary personnel with broad knowledge. In addition, the availability of already existing literature should be evaluated. A scoping review was performed. Methodological, empirical, review, and theoretical articles were evaluated. Only a minority of the studies (1.3%) evaluated motivational theories in veterinary health care. This reflects that there is a need for research in this field. Still, it is illustrated that the veterinarian profession would benefit from the implementation of general psychological as well as motivational work theories.

## 1. Introduction

Motivation derives from the Latin term “movere” (to move). Motivation is one of the most important reasons for individuals to move forward to acquire a specific goal [1].

In classical literature, the individual’s concerns about motivation are strictly separated into aims one wants to achieve in the workplace and aims one wants to achieve in private life. This resulted in the fact that the motivation of the individual in their personal life and the motivation of the performance of employees have long been seen and referred to as two independent topics. This view conflicts with the fact that work psychology is applied psychology [2]. In 2001, the journal “Academy of Management Review” called for articles on the future of work motivation. Researchers submitted fifty-six documents in response to the call. What these publications have in common is a sincere desire to improve upon current theories of work motivation by modifying and extending them to reflect contemporary working conditions. An increasingly short-term orientation describes modern workplaces, where time is a critical performance variable and there is increasing interdependence between staff, evolving affective responses to the workplace experience, rising value and motive conflicts among employees, and a clear recognition of the transient nature of careers [3]. This steers us towards more multidisciplinary research, considering general psychology, work psychology, and various other factors to extract all possible dimensions of motivation [2].

This multidisciplinarity in evaluating one’s motivation is vital in every profession. Still, it is of extraordinary benefit in healthcare professions, as they often lack a clear separation between private and professional lives. In particular, the veterinary sector is characterized by some peculiarities compared to other domains: career choices are mainly based on natural affection for the occupation and the love of animals [4]; veterinary professionals describe long working hours, an excessive workload, and the feeling of too much job involvement [5]. This, combined with the fact that veterinarians, especially in equine work, experience a considerable number of physical (serious) harms during their career [6], could be a possible explanation for the high percentage (up to 15%) of burnout among professionals and a commensurate percentage of suicides around four times greater than that of the public [7]. Many nations, including the United Kingdom, the United States, Canada, and Norway, have developed stress management strategies, such as a 24-hour peer-support telephone hotline service, programs of mental health lectures in veterinary schools, and training modules on vital professional skills [8].

Although these interventions certainly have a positive influence, the primary goal should be to adapt the essential characteristics of the veterinary profession by identifying the most significant challenges the profession faces and possibilities to address those to improve well-being at work in the veterinary profession. For evaluation and possible improvement of employees’ well-being, employees themselves as well as the managing staff must understand every individual´s motivation. Defining the motivation of veterinary professionals in a multidisciplinary way can be a first step in identifying stressors within the general profession and within individual workplaces. Adapting their characteristics can lead managing staff to suitable individualized prevention plans and can be the basis of implementing functional (individual) management systems addressing negative peculiarities, as well as increase well-being within individual workplaces and within the profession in general. This can be achieved by providing direction and guidance to not only researchers but also to the profession itself in the future.

Knowledge of classical general psychologic motivational theories and work motivational theories are required to build on existing theories of work motivation and adapt and extend them to fit into the veterinary profession. This article therefore first provides an overview of the theories before discussing the advantages and disadvantages of these as a driver for workplace health promotion, employees’ well-being, and quality of life within the veterinary health care sector. This scoping review therefore first systematically maps general research done in this area to provide an overview of the theories, before discussing the advantages and disadvantages of these as a driver for workplace health promotion, employees’ well-being, and quality of life within the veterinary health care sector. 

## 2. Material and Methods

In conducting this scoping review, we followed the Preferred Reporting Items for Scoping Reviews and MetaAnalyses model according to PRISMA [9]. According to Trico et al., scoping reviews can study the scope and size, the range and variety, and the nature or characteristics of the evidence on a subject or question. It can sum up outcomes from a body of knowledge which is varied in methods or discipline or identify special gaps in the literature to support future research [10]. The search was performed in Pubmed, Web of Science, Scopus, and Google Scholar. The search strategies and terms were defined in a team discussion all authors attended: as search terms, “motivation” “motivational theories”, “workplace”, “employees”, “(veterinary) health care”, and their German equivalents were used. The scientific quality of the publications was assessed. Content analysis using factors affecting motivation within the health care profession and potential benefits of using motivational theories in the health care sector and veterinary health care sector was performed. The authors included review papers, methodological studies, and theoretical articles. Review papers were included if they were written in English or German and gave an overview about specific motivational theories or their use in the occupational world. Different types of methodological studies in English and German language were included to consider different aspects of measuring motivation in (health care) employees. Theoretical papers written in English or German were included if they contributed to the understanding of the concept of motivation theory or its application in the professional (health care) world. Records were excluded if they did not meet the criteria or did not fit in the conceptual framework of the study, were published in a language other than English or German, or were editorials, letters, commentary, press releases, and grey literature. To secure consistency among the reviewers, all of them evaluated the 65 publications included. Evaluation was followed by discussion of the results. Disagreement on publication inclusion was solved by discussion within the reviewer team. A data table was developed by one of the reviewers to assign the various publications to their respective motivational theories. The table was discussed and adapted within the whole reviewer team. Within each motivational theory, the methodological studies were evaluated in terms of the population studied, the settings, and the study design. Systematic reviews were examined for the number of included studies that might have met our inclusion criteria to determine the number of studies that were omitted from our search.

Table 1 and Table 2 provide an overview of the inclusion and exclusion criteria.

## 3. Results

**Figure 1:**
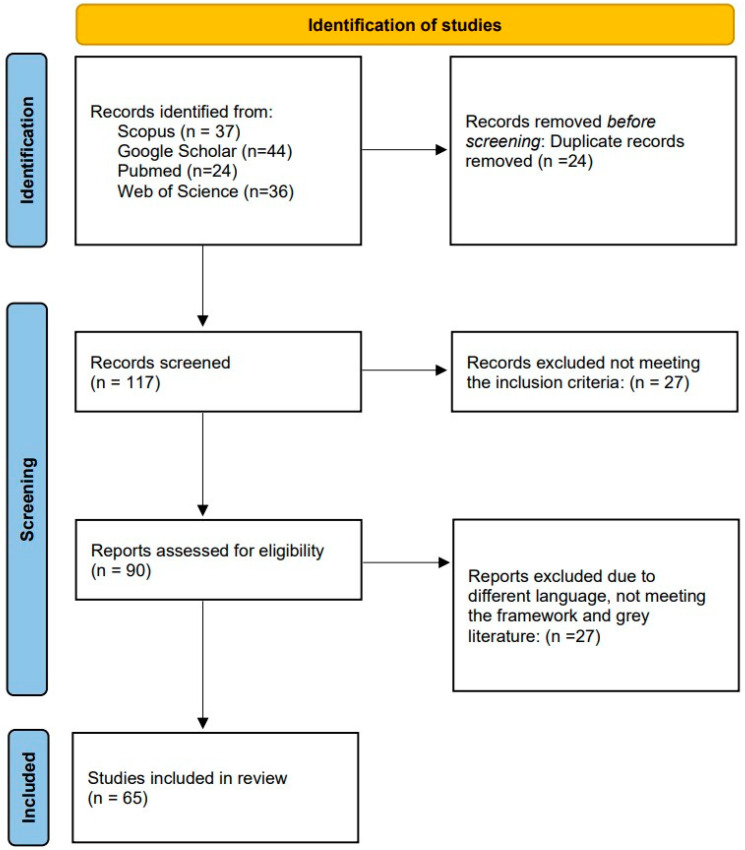
Identification of studies.

Initially, 141 articles were identified by title, of which 86 abstracts and 54 full-text articles could be evaluated regarding the aspects mentioned earlier. For the present review, 65 publications, 15 methodological studies, 14 review articles, and 36 theoretical articles were assessed (see Supplementary Materials). Figure 1 shows the evolution of the number of papers during the inclusion process.

The corresponding table with the overview of the reviewed articles can be found in the Supplementary Materials.

The following sections will provide an overview of motivational theories in general and their use in today’s organizational world.

### 3.1. Motivation in General Psychology

Motivation refers to behavior initiation, direction, intensity, and persistence in psychology. Motivation is a temporal and dynamic state that should not be confused with personality or emotion [11]. Psychology has numerous approaches to explain motivation.

#### 3.1.1. Hedonism

Psychological hedonism dates back to Democritus and Epicurus (460 BC). The philosophers Locke, Bentham, Mill, and Helvetius discussed hedonism in the seventeenth and eighteenth centuries [3], and research about psychological hedonism is still an ongoing topic.

“Hedonism” is derived from the ancient Greek term for pleasure. Psychological or motivational hedonism asserts that only pleasure and suffering motivate the individual. Pleasure is equated with all pleasant sensations, while pain includes all aversive feelings. All other desires are seen as purely instrumental concerning these two ends. As psychological hedonism defines pain and pleasure as sources of motivation, determining one’s behavior will sometimes fail due to factors outside one’s control or the “weakness of one’s will”. This reflects that psychological hedonism does not account for whether a person will succeed in achieving any goal in their behavior [12,13,14,15]. More recent studies discuss the conceptual problems of hedonism: the ubiquity of counterexamples, the inability to test the theory, and the lack of plausible mechanisms have been pointed out in numerous publications [16].

#### 3.1.2. Instincts

Behavioral scientists like James, Freud, and McDougall proposed more empirical-based problems to explain motivation. McDougall described “instinct” as “an inherited or innate psychological predisposition that determines its possessor to perceive, or pay attention to, objects of a particular class, to experience an emotional excitement of a particular quality upon perceiving such an object, and to act in a specific way in relation to it.” [17]. The general idea is that similar motivations occur in humans because of the parallel biological programming humans share. Locomotion, curiosity, sociability, fear, jealousy, and sympathy were identified as such instincts [18].

Increasing limitations of the approach began to appear, and the instinct theory began to be replaced by models based on drive or reinforcement, given that this theory argues that the source of our motivation is biologically or genetically programmed into our unchanging body [3]. This conflicts with, e.g., learned human behavior. Nowadays, psychologists are beginning to believe that while instincts can be programmed genetically, their manifestation is also influenced by individual experiences [18].

#### 3.1.3. Drive Reduction Theory

The drive reduction theory of motivation evolved in a prevalent way to explain motivation during the 1940s and 1950s. The theory was first created by the behaviorist Clark Hull. In accordance with the theory, the primary force behind motivation is the reduction in drives. Hull’s theory is based on the concept of homeostasis, which states that the body actively works to maintain a specific state of balance or equilibration. It was believed that behavior was one of the ways that an organism maintains this balance. Drives like thirst, hunger, and the need for warmth, also called primary drives or reinforcers, create an unpleasant state and tension that needs to be reduced. Hull proposed that humans and animals mimic any behavior that decreases these drives. Reduced drive acts as a reinforcement for that behavior. Whereas primary drives are directly linked to survival, secondary or acquired drives, such as the desire for money, intimacy, or social approval, are socially specific or learned [19,20,21].

Hull’s drive reduction theory lacks generalizability, so it was criticized soon after publishing. Yet, most motivational theories that arose in the 1950s and 1960s were either based on Hull’s original idea or focused on offering alternatives to the drive-reduction theory. A notable example is Abraham Maslow’s famous hierarchy of needs, which evolved as an alternative to Hull’s approach and is still utilized by managers in many businesses [22].

A fundamental criticism of the drive reduction theory is that it fails to account for how secondary drives or reinforcers lower an individual’s drives. In contrast to primary drives, secondary reinforcers have no direct effect on physiological and biological demands. Wealth is a typical example of a secondary drive. Money enables the acquisition of primary reinforcers but has little effect on reducing urges. Nevertheless, it serves as a potent source of reinforcement. Another significant criticism of drive reduction is that it does not explain why people engage in actions that do not diminish their drives or why specific drives cannot be met yet appear to increase tension, such as extreme sports [2,3].

#### 3.1.4. Arousal Approaches

Arousal theory extends drive-reduction theory by considering levels of “arousal”, which in psychology represents mental alertness, as potential motivators. People have varying levels of optimal arousal and are motivated to take actions that will help them achieve their optimal level. Assume an individual’s arousal level falls below the optimal threshold, resulting in demotivation until the arousal level rises again. Simultaneously, if an individual’s mental alertness becomes too high, they will become demotivated until their arousal level returns to the desired level. However, studies show that moderate arousal levels result in the most motivation [23,24,25,26]. Within the arousal theory, the Yerkes–Dodson Law exists. According to this hypothesis, a state of optimal arousal results in optimal performance. Too little arousal does not provide much in the way of motivation, but too much arousal generates mental overstimulation. It can cause an intense stress reaction, decreasing performance and motivation [27].

#### 3.1.5. Cognitive Approaches

The cognitive approach revolutionized psychology in the late 1950s and early 1960s. Interest in mental processes has been slowly rejuvenated via the engagement of Piaget and Tolman [28,29]. Cognitive psychology is the science of how we think. Cognitive motivational approaches try to explain an individual’s behavior by evaluating one’s thought processes and the deliberations towards a decision about a specific activity by drawing a line between intrinsic and extrinsic motivation and examining the effect of these on the individual [2].

Intrinsic motivation encompasses all the variables that motivate an individual based on internal rewards, such as self-improvement or assisting a friend in need. Learning valuable skills may motivate an employee to seek a promotion. In this case, the theory would refer to a positive intrinsic motivation drive. If an employee wants to learn new things because he or she is dissatisfied, negative intrinsic motivation is the driving force. The actions still have a positive outcome, but the motivation used is aimed at preventing a negative outcome rather than creating a positive outcome. However, whether positive or negative, intrinsic motivation is usually more sustainable than extrinsic motivation because it focuses on positive or altruistic things that one can debate [30,31,32,33].

Extrinsic motivation, on the other hand, typically focuses on things that are given to you by someone else and thus are not directly within your control to achieve [31]. Extrinsic motivation refers to all the factors that motivate a person based on external rewards such as money, praise, and accomplishing goals due to a tangible incentive, fear, or expectation, all of which are dependent on external factors. These types of motivators are more common than intrinsic rewards. Because of the anticipated raise, an employee may wish to be promoted. Extrinsic motivation, like intrinsic motivation, can be negative; for example, if an employee is motivated to perform better because they are afraid of losing their job [34].

Comprehensive research was conducted to investigate if extrinsic motivation may produce the same levels of satisfaction and psychological well-being as intrinsic motivation. The dispute is ongoing, but data indicate that intrinsic motivation supports psychological well-being more than extrinsic motivation [35]. Following this, intrinsic motivation factors have also been identified to be of importance within the veterinary profession. Probably without the researchers knowing that they were operating within the cognitive theory of motivation, they proved that having high control [36], high satisfaction [37], and high work commitment [38] mitigate the effects of stress using a survey among veterinarians [39]. In 2008 it was again indirectly proved that cognitive motivational approaches have their place in the veterinary profession. A Belgian study on stress, work–home interference, and burnout among Belgian veterinarians referred to a transactional model [40,41] based on cognitive theories and coping strategies, emphasizing the relationship between environmental demands and human reactions [5,42].

While publications regarding rewards within the veterinary profession are few and far between [43], intrinsic and extrinsic motivators are the subject of some publications within the human healthcare sector [44,45,46] and have often been studied in entirely different sectors [33,47,48]. Classical work psychology defines rewards as being either intrinsic or extrinsic. Intrinsic rewards stem from rewards that are inherent in the job itself. The employee enjoys them due to successfully completing the task or attaining goals. Extrinsic rewards are those that are external to the task of the job, e.g., salary, work conditions, fringe benefits, security, and promotion [49]. This separation could be unclear when the theory is applied to the veterinary healthcare sector with its special peculiarities (see later). Only one single publication dealing directly with cognitive motivation theory within the veterinary profession could be found: Driscoll (2022) indicated in his literature review that veterinary technicians’ intrinsic and extrinsic rewards are complex [43].

### 3.2. Motivation in Work Psychology

Work psychology defines motivation as a collection of energetic powers that arise within and beyond a person to initiate work-related behavior and define its form, direction, vigor, and duration [31].

Various views furnish a framework of how best to motivate employees to work willingly and effectively and deliver a foundation for reanalyzing the most influential motivational style [50]. Motivational theories in work psychology are mainly divided into two approaches: content theories, based on the early ideas on work motivation, and process theories [51].

#### 3.2.1. Content Theories

Content theories emerged during the 1950s. Since those theories attempt to explain the specific things which motivate the individual at work, they are collectively referred to as content theories. Major content theories of work motivation include:

1. Maslow’s hierarchy of needs theory;
2. Alderfer’s modified need hierarchy model;
3. Herzberg’s two-factor theory;
4. McClelland’s achievement motivation theory.

##### Maslow’s Hierarchy of Needs Theory

Maslow’s (1954) needs hierarchy system is a widely used scheme for categorizing human motives [52]. Maslow’s basic proposition is that people are wanting beings who, as they grow, work their way up a hierarchy based on meeting a set of prioritized needs. Individuals, according to him, always want more, and what they want is determined by what they already have. In most cases, the hierarchy is depicted as a pyramid with five main levels. The lowest level includes physiological requirements. The necessity for self-actualization constitutes the highest level [50]. Individuals move up the hierarchy as each lower-level need is met. According to Maslow, the first three needs are deficiency needs that must be met before individuals can develop a healthy personality. The final two needs are related to individual achievement and the development of human potential. Maslow states that unsatisfied needs motivate people, so once a lower need is met, it no longer acts as a strong motivator [52]. The need hierarchy model is still applied today by many managers as a good base for evaluating motivation at work. While some needs are manageable and affordable to execute, others are complex and pricey [53,54].

##### Alderfer’s Modified Need Hierarchy Model

Alderfer modified Maslow’s hierarchy of needs model in 1972 to include only three needs: existence, relatedness, and growth (ERG theory). Existence must meet material nature’s physiological and safety needs. Love or belonging, affiliation, and meaningful interpersonal relationships of a safe or esteemed nature are all examples of relatedness. Self-esteem and self-actualization must be addressed as part of the growth process [55].

According to Maslow, individuals evolve through the hierarchy from existence needs to relatedness needs to growth needs as lower-level needs are met. In contrast to his predecessor, Alderfer believes that these needs are more of a continuous spectrum than hierarchical levels, and that multiple needs can be activated at the same time. This implies that if one’s needs are blocked at a particular level, then attention should be focused on satisfying needs at the other levels. Moreover, people may also progress down the hierarchy [55].

##### Herzberg’s Two-Factor Theory

Herzberg expanded on Maslow and Alderfer’s work by attempting to comprehend how work activities and the nature of one’s job influence motivation and performance. He established the motivation-hygiene theory, also recognized as the two-factor theory of motivation. Herzberg performed a widely publicized motivational study on 200 accountants and engineers working for companies in and around Pittsburgh, Pennsylvania. Employees were asked to recall instances when they felt particularly good or bad about their current or previous jobs. Herzberg asked the participants to provide reasons for their feelings and explain the sequence of events that led to them. According to Herzberg, reported good feelings were generally associated with job experiences and job content, whereas reported bad feelings were typically associated with the job’s surroundings and job context [56,57].

Herzberg stated that job satisfiers, referred to as motivators, are connected to job scope and that job dis-satisfiers, referred to as hygiene factors, are related to the context of the position. The term hygiene refers to factors that are preventive, as they are those factors that prevent dissatisfaction. Herzberg saw these hygiene factors as being far more secular, directing to satisfaction and future motivation [58].

Herzberg’s theory is now regarded as one of the most empirically sound and compelling theories of motivation. It is the only theory distinguishing between motivating and demotivating factors [52]. Herzberg is credited with being the first to introduce the field to the role of job design, specifically job enrichment, as a critical factor in job motivation [3].

Several studies investigating job satisfaction in human medical nursing populations have incorporated Herzberg’s theory, and numerous others have also used it as a conceptual framework [56]. In their study of job satisfaction among 147 nurse practitioners in the United States, Cancel and colleagues (2005) utilized Herzberg’s theory as a framework. This quantitative descriptive study discovered that motivation and hygiene contributed to job satisfaction. In addition, the authors noted that boosting hygienic elements, particularly pay and benefits, increased job satisfaction [59].

Lephalala (2006) utilized Herzberg’s theory to investigate the factors influencing the turnover of 136 nurses in private hospitals in England. Motivational factors were identified as impacting nursing turnover and unhappiness. In addition, hygiene problems contributed to nurses’ discontent with compensation and management policies [60]. Mitchell (2009) investigated job satisfaction and burnout among 453 Saudi Arabian nurses with international training. The foundation of the research was Herzberg’s hypothesis, which revealed that motivation and hygienic aspects influenced job satisfaction. These elements were work recognition, salary, working conditions, accomplishment, corporate policy and administration, and connections with managers and coworkers. Yet, they ascribed employment discontentment to hygienic issues, such as business policy and administration, working circumstances, status, supervisor relationships, security, and personal life [61].

##### McClelland’s Achievement Motivation Theory

In 1961 McClelland introduced his developed needs theory in the book ‘The Achieving Society [62]. Individuals’ specific needs, according to this theory, develop over time and are shaped by their life experiences. He discovered three major developed motives that influence a person’s motivation and effectiveness in specific job functions:

- Achievement motive (n-ach)

The n-ach person is high in the need to achieve and is predisposed to striving for success. This person is highly motivated by attaining realistic but challenging goals and advancement in the job. There is a strong need for timely criticism and feedback about achievement and progress and a need for a sense of accomplishment. High n-ach people prefer tasks with a moderate probability of success, ideally a 50% chance. They prefer either to work alone or with other high achievers.

- Authority/power motive (n-pow)

N-pow individuals are “authority motivated”. They must influence others directly by making suggestions and giving their opinions and evaluations. A solid need to lead and to prevail in ideas is typical, as well as motivation and the need to increase personal status and prestige. An N-pow individual with a high need for power but a low need for warm, supportive relationships might become dictatorial. In contrast, one with an increased need for friendship might become a social worker.

- Affiliation motive (n-affil)

The n-affil individual requires positive relations and is driven to maintain strong, warm interactions with others. They are motivated by the desire to have harmonious relationships with others and to feel accepted by others. These individuals are team players who are frequently agreeable and provide emotional support to others. The n-affil employee excels at customer service and client interaction.

McClelland remarked that individuals maintain and show a mix of these characteristics. At work, his motivational or needs mix, which consequently affects and characterizes their behavior and working and managerial style. This implies that people with different needs are motivated differently [53,62,63].

McClelland’s concept is an appropriate source of motivation for the modern world since it accounts for variables such as cultural issues outside the business, which can influence individual behavior. As Maslow, Alderfer, and Herzberg never investigated those factors, McClelland’s theory differs crucially from the other content theories [64].

#### 3.2.2. Process Theories

In contrast to prior content theories, process theories aim to establish the links between the dynamic factors that comprise motivation and the actions necessary to impact behavior. These ideas focus on how behavior is begun, guided, and maintained over time and events. Prominent motivational process hypotheses include [2]:

1. Expectancy-based models: Vroom and Porter and Lawler;
2. Equity theory: Adams;
3. Goal theory: Locke;
4. Attribution theory: Heider and Kelley.

##### Expectancy-Based Models: Vroom and Porter and Lawler

The expectancy theory-based models are generic theories of motivation and are not developed by a sole author. Vroom (1964) and Porter and Lawler (1968) worked on more recent approaches to the expectancy theory. The underlying basis of all expectancy theories is that the expected results of their actions influence people. Motivation is determined by the relationship between effort exerted and perceived level of performance, the anticipation that rewards will be related to performance, and the assumption that rewards will be available [51].

The goal of Victor Vroom’s expectation theory of work motivation is to provide an answer to the question of what determines an individual’s willingness to devote personal effort to tasks that contribute to the performance of the work unit and the organization. He argued that individual qualities such as personality, skills, knowledge, experience, and abilities determine an employee’s performance. According to him, effort, performance, and motivation are all tied to an individual’s motivation. The variables Expectancy (E), Instrumentality (I), and Valence (V) are used. Vroom hypothesized that the following equation relates these variables and motivation, (M): M = E × I × V [15].

This equation shows that motivation is sharply reduced whenever one or more factors approach zero value [51].

Porter and Lawler expanded upon Vroom’s idea of expectation. They recognized, unlike Vroom, that exertion does not immediately correlate with performance. Actual performance is mediated by a person’s abilities and characteristics, as well as their role perceptions. In their hypothesis, rewards are inserted as an intervening variable. They view motivation, satisfaction, and performance as distinct variables and strive to explain their intricate interrelationships. They concluded that job satisfaction depends on performance more than performance depends on job contentment [65]. Even if the theories are still applied in today’s organizational world, they have been criticized for their high degree of rational recognition of individuals [66].

##### Equity Theory: Adams

In 1963, Stacy Adams introduced the equity theory to explain how employees respond to perceived unfairness in the workplace. His work concentrates on people’s perceptions of how fairly they have been treated compared to colleagues. An individual perceiving inequity will develop tension or drive and be motivated to reduce or eliminate the inequalities [67].

An individual’s input and outcomes are determined by their perceptions. Age, sex, education, social standing, organizational position, credentials, and effort affect input variables. Output variables include compensation, status, promotion, and intrinsic interest in the job [67].

Recent publications address the equity theory in human health care: a Dutch study among 567 general human practitioners examined the connection between harassment by patients, emotions of inequity, and social support for burnout. They stated that burnout consists of two dimensions: emotional exhaustion and negative attitudes towards one’s patients and towards oneself, e.g., depersonalization and reduced personal accomplishment; that feelings of inequity and social support play an intervening role between harassment by patients and emotional exhaustion; and that emotional exhaustion fosters a negative attitude, which in its turn aggravates the doctor’s poor relationships with their patients. The Dutch authors concluded that equity theory enhances understanding of the relationship between practitioner and patient as a determinant of burnout [67]. No veterinary publications could be found.

##### Goal Theory: Locke

In the late 1960s, goal-setting theories emerged, as it was discovered that setting targets for behavior improved task performance [3]. One of the first theories was published by Locke [68]. Locke presumed that once an individual opts to pursue a goal, they will regulate their behavior to achieve it. The interaction of goal specificity, goal difficulty, and the extent of a person’s goal commitment governs the effort expended and improves task performance [51,69].

Managers believe that the level of effort expended is regulated by the combination of goal difficulty and the extent of the person’s commitment to achieving the goal. Individuals with specific quantitative goals, such as a fixed timeframe for finishing an assignment, outperform those with no set goal or only an unclear goal. Furthermore, it is believed that people who set challenging goals outperform those who set more manageable goals [51]. In 2009, it was stated that using the idea in a specific hospital enhanced job motivation when nurse managers had a clearly defined objective for nurses [70].

##### Attribution Theory: Heider and Kelley

In contrast to alternative theories, the attribution theory focuses more on the relationship between personal perception and interpersonal action than on individual motivation. Fritz Heider believed in 1958 that internal and exterior influences contribute additively to behavior determination. Internal factors include personal characteristics such as ability, effort, and exhaustion, whereas external forces include environmental characteristics such as rules and the weather. Heider emphasized that perceived, not actual, factors are crucial to behavior and that people would respond differently depending on whether they perceive internal or external characteristics [71].

Harold Kelley, a prominent theorist, noted in 1973 that attribution theory is primarily concerned with the cognitive processes through which an individual perceives his or her conduct as being produced by certain aspects of the relevant environment. Though most factors are not clearly observable, the theory emphasizes that humans must rely on cognition. Individuals are supposed to be rational and motivated to recognize and comprehend the causal structure of their relevant environment [72].

The attribution theory describes work motivation based on the locus of control, or whether personal views results to be controlled by themselves or external causes.

According to Kelley, three crucial factors interact to determine whether an internal or external attribution is selected: uniqueness, agreement, and consistency. When they see low distinctiveness, low unanimity, and high consistency, individuals link behavior to internal forces or personal factors. When people sense strong distinctiveness, high unanimity, and soft consistency, attribution to external forces or environmental causes will occur [50,71].

The fact that there is an increasing variety of attribution theories reflects the importance of this theory in modern work structures [73]: more recent studies considering the attribution concept view individuals as active interpreters of their life events. In their interpretations, they employ consistent and rational ways of meaning construction. The more crucial or unexpected the event, the greater the likelihood that individuals will seek an explanation to make sense of it, typically by identifying its source. Every statement an individual makes and every action an individual performs can be subject to attributional analysis, not only by the person but also by others. The outcome of this analysis has significant implications for how people think about one another and how we respond to another’s actions. Whether it is an accomplishment, failure, a stigmatizing condition, a need for help, or an aggressive act, if these are attributed to controllable and intended causes, for instance, responses of anger and reprimand or neglect are more likely [74]. In contrast, uncontrollable and unintentional attributions are more likely to lead to compassion and an offer of support.

## 4. Discussion

Going through the different motivational theories, one will have recognized several practical and less applicable aspects of each theory. Evaluating them from a critical point of view will be helpful to eventually implicate them into the functional framework of the veterinary profession.

The concept of psychological hedonism is intuitively appealing and can explain some behavior patterns. Regarding the veterinary profession, it is challenging to clarify the voluntary willingness to adapt to the unique characteristics (career choice made based on affection, long working hours, high workload, physical hazards) of the profession to a certain point. Nevertheless, one should respect this theory since advances in affective neuroscience and psychology provide the foundation to reformulate the theory to overcome these problems. Further research is ongoing and will eventually benefit the veterinary sector in the future [15].

As already described earlier, instinct approaches cannot clarify all aspects of human motivation. Considering veterinary health care, it is doubtful that instincts are responsible for resuming work after, e.g., physical trauma. It would be more likely that people would stay away from dangerous work from the outset. But, as research regarding the influence of experiences on instincts is still ongoing, the profession should refrain from negotiating knowledge of this theory [18].

At first glance, Hull’s drive reduction theory seems to be worthless for veterinary medicine. Or how should one, based on this theory, explain why individuals seek a profession like veterinary medicine that often interferes with fulfilling biological needs, e.g., lack of sleep or irregular food consumption, and which places them at notable risk? Logically, the drive reduction theory cannot account for such behaviors. But, in this context, another question arises: is one aware of the specific characteristics of the profession when taking the first steps into a veterinary career, and is one faced with hazards that have never been considered before when entering the profession? Or, is the interference of fulfilling biological needs an essential factor for many employees to leave the profession? From this point of view, Hull’s drive reduction theory should not be disregarded entirely. It should rather be considered when it comes to educating students regarding the peculiarities of the profession, and it should be the basis for investigating why practitioners leave the profession.

As mentioned, the arousal theory develops upon the drive reduction theory by considering “arousal” levels. If the arousal level is too low, actions will be taken to raise it; if it is too high, reductions will follow. Although this theory does not explain working hard for a degree in vet school, this theory could be of benefit to the veterinary profession: appplying the Yerkes–Dodson Law to the veterinary sector means that every veterinary employee experiencing too much job involvement can end up in overstimulation and burnout. To combat high levels of job involvement, one must lower the arousal back to optimal levels by having regulated off-times [26]. Motivation is about balancing high stimulation levels and rest and recovery periods. To incorporate knowledge and a sense of the importance of this theory into the thinking of the whole profession would be a big step in the right direction. Therefore, the theory must not be dropped as useless for the profession.

Cognitive motivational approaches explain one’s behavior based on intrinsic and extrinsic motivation [2]. Intrinsic and extrinsic motivators have been the subject of some publications within the human healthcare sector [44,45,46] and have often been studied in entirely different sectors [33,47,48]. Indirectly, as described above, intrinsic motivation factors have already been identified to be of importance within the veterinary profession: recent publications proved that having high control [36], high satisfaction [37], and high work commitment [38] mitigate the effects of stress [38]. Furthermore, a veterinarian study on stress, work-home interference, and burnout referred to a transactional model [40,41] based on cognitive theories and coping strategies, emphasizing the relationship between environmental demands and human reactions [5,42].

As mentioned earlier, classical work psychology defines rewards as being either intrinsic or extrinsic [49]. When investigating the veterinary sector, which is characterized by its previously described peculiarities like the lack of a clear separation between private and professional lives, where career choices are mainly based on natural affection for the occupation and the love of animals [4], where long working hours, excessive workload, and the feeling of too much job involvement is present [5], a clear separation between intrinsic and extrinsic rewards seems to be past the reality. This could be a hurdle when it comes to applying the theory in veterinary medicine and could be a reason why the author could find only one single publication that deals with it directly: Driscoll (2022) indicated in his literature review that veterinary technicians’ intrinsic and extrinsic rewards are complex [43]. Nevertheless, even if this first publication reports complexity, it should be seen as a future guideline to build on for the profession.

Maslow’s hierarchy of needs theory considers individual needs as being organized hierarchically, beginning at the bottom of a pyramid. As each need is satisfied, other needs arise. Physiological needs form the lowest part of this hierarchy, followed by safety, belongingness/love, esteem, and self-actualization (highest). As described before, managers in all professions still apply Maslow’s theory, with some organizational factors being manageable and affordable to execute. In contrast, others are more complex and pricier [53]. Veterinary medicine is uniquely positioned to apply these organizational factors compared to other professions. While most jobs will not interfere with physiological needs (lowest level), within the veterinary sector, one’s professional life often interferes with food intake and sleep. Considering the high incidences of work-related accidents, the veterinary profession also collides with Maslow’s second level (safety). As Maslow asserted the first three needs represent deficiency needs that must be mastered before an individual can develop a healthy personality, implementing Maslow’s theory would undoubtedly have a positive impact, but only after adapting the essential characteristics of the veterinary profession and enhancing the well-being of veterinarians at work.

The ERG theory of Alderfer reduces the five demands to three: existence, relatedness, and development. Individuals can engage multiple needs simultaneously and proceed down the hierarchy. Alderfer, unlike Maslow, maintains that lower level wants only need to be met after a higher-level need might arise as a driving factor. So, if an individual’s yearnings at a certain level are obstructed, he/she will focus on satisfying needs at other levels [51]. Per Maslow’s theory, Alderfer’s model is still applied by many managers in the workplace. The ERG theory indicates that managers must identify their employees’ simultaneous needs. Focusing exclusively on one need at a time will not motivate employees [53]. For the veterinary profession, this would mean that sleep deprivation and irregular working hours could be compensated by, e.g., optimal supervision, efficient training, or high job status. Further research is necessary to identify the most critical “motivating factors” within the veterinary profession.

In worldwide research, the two-factor theory of Herzberg remains to determine and identify the level of job satisfaction. The fact that several studies investigating job satisfaction in human medical nursing populations have incorporated Herzberg’s theory, and numerous have also used it as a conceptual framework [57], is promising for the veterinary profession. Human medicine studies already discovered that motivation and hygiene contributed to job satisfaction and that boosting hygienic elements, particularly pay and benefits, work recognition, working conditions, accomplishment, corporate policy and administration, and connections with managers and coworkers increased job satisfaction [59,61]. Employment discontentment with hygienic issues, such as business policy and administration, working circumstances, status, supervisor relationships, security, and personal life, was also described [61]. Human medicine also uses Herzberg’s theory to investigate another problem with which veterinary medicine also struggles: turnover in hospitals. As described, higher motivational factors were identified to impact nursing turnover, unhappiness, and hygiene problems, which contributed to nurses’ discontent with compensation and management policies [60].

The human health sector findings suggest, with an almost certain probability, that Herzberg’s theory could also be of value to the veterinary profession. Even if we do not use these studies to conduct studies within our profession, the results may still be actionable for veterinary managers and employees as well.

As previously mentioned, McClelland’s concept is an appropriate source of motivation for the modern world since it accounts for variables such as cultural issues outside the business, which can influence individual behavior [62]. Dealing with different cultural aspects and individual behavior brings us back to applying general psychology in the workplace and again implies the need for multidisciplinarity within future research.

Expectancy theories are still applied today. According to the theory, employees value anticipated corporate prizes. To strengthen the performance–outcome link, managers should implement mechanisms that closely link rewards to performance. A crucial factor often missed is ensuring that the provided tips are deserved and wanted by the recipients [37]. The theories assume that humans maximize utility and avoid pain, prioritize clearly, decide on their workload, and cherish rewards. The reality is different, and the theories have been criticized for their high degree of rational recognition of individuals [66]. But, keeping in mind that generalizing a particular profession and neglecting people’s individuality, the veterinary profession would benefit from further research regarding preferred rewards.

As per Adam’s equity concept, individuals who sense inequity will attempt to decrease it by cognitively distorting inputs and results (“cognitive distortion”), modifying inputs and outputs directly, or leaving the firm. Adam’s idea has far-reaching ramifications for staff morale, productivity, efficiency, and turnover [53].

As mentioned earlier, the equity theory has already been the subject of several studies in the human healthcare sector. Among other things, the studies addressed burnout, stating that equity theory enhances understanding of the relationship between practitioner and patient as a determinant of burnout [68]. As the veterinary profession is faced with a burnout incidence of up to 15% and suicide rates four times higher than public rates [7], it would be exceedingly negligent to ignore this theory. Applying the results of the human setting would help. But, using those studies to set up veterinary studies should be the ultimate goal.

According to its creator Edwin Locke, the goal-setting theory is more a method than a traditional theory. Employee motivation will be optimal specific, measurable, and attainable, and time-related goals are set in collaboration with the employee. While feedback is seen as critical in all cognitive theories to a different extent, the goal-setting theory emphasizes feedback more clearly. This is one of the advantages of the theory, as an understanding of outcomes allows the individual to improve. Recent studies discuss implementing the goal-setting theory within human hospitals, consistent with the other process theories: job motivation was enhanced when nurse managers had a clearly defined objective for nurses [68]. Furthermore, results from health service studies showed that the major factors affecting job satisfaction and causing dissatisfaction were workload, promotion, health and safety, relationships with supervisors, as well as recognition and appreciation [75].

Unfortunately, as the authors know, similar studies for the veterinary healthcare sector are missing. The fact that there is an increasing variety of attribution theories reflects, as previously mentioned, the importance of this theory in modern work structures [73]. To the author’s knowledge, no studies have been conducted to date within veterinary medicine dealing with the subject. On the one hand, this should be alarming, on the other hand, this leaves room for future studies.

### 4.1. Conclusions

It can be concluded that, disregarding hedonism and instinct theories, motivational theories in general psychology could benefit the veterinary profession. While the cognitive and motivational approaches are the subject of ongoing research, Hull’s drive reduction theory and the arousal theory can be considered basic needs the veterinary sector must meet. Thinking about the Yerkes–Dodson Law helps us to be aware that the profession should strive for the best possible level of mental alertness to achieve optimal motivation for its employees. The drive reduction theory illustrates straightforwardly the importance of having respect for rest periods, a regulated shift system, and good training to avoid traumatic and psychological risks, which could very likely be the first steps in increasing the motivation and well-being of veterinary employees. This is the basic but probably most crucial conclusion veterinary managers and employees can draw from this article. Without the fulfillment of those basic needs, an adaptation of the specific negative characteristics and the associated impact on individuals’ motivation and health of the profession will not be possible. Applied within veterinary medicine, this means that the individual employee must, often against the specific character traits that many veterinarians bring with them, raise awareness regarding the specific hazards affecting their health. Individuals must be willing to protect themselves by, for example, demanding a regulated shift system and by, for example, paying attention to a good work-life balance. Elder, experienced staff can have an influence on younger employees and act as a positive, but of course also as a negative, example.

At the same time, however, managing staff must create an environment in which it is possible to satisfy these basic needs by, e.g., providing state-of-the-art equipment and good training to minimize their employees’ risk of being injured. Also, management should be aware that a regulated shift system is a basic need. However, shift systems can only be functional if a certain number of staff are employed. Sick leave, vacation, and compensatory time off must be considered, and the number of employees may need to be adjusted.

For researchers, especially cognitive motivational approaches, could provide direction and guidance in establishing rewards in this special profession. Concerning the content theories, at least Maslow, who stated that the first three needs of his pyramid represent deficiency needs that need to be mastered before individuals can develop into a healthy personality, connects to the context of adapting the essential characteristics of the veterinary profession and improving well-being within the veterinary profession. Alderfer’s theory differs slightly from this because the different stages can be reached in any direction by introducing “motivating factors” compensating for negative aspects. Indeed, the sector would benefit from future research regarding these factors. However, adapting to the negative characteristics of the industry should be the first goal for the profession. The numerous publications discussing Herzberg’s theory in the context of the human healthcare system implicate the need for research regarding content theories in our sector.

Furthermore, it can be concluded that the human healthcare sector already benefits from implementing different process theories. This means that, especially for veterinary companies in which the managing staff have already addressed the basic needs described above, there could be a benefit in applying those theories as a means of improving employees’ well-being and quality of life.

Managers knowing and applying the expectancy theories could strengthen the performance–outcome link by implementing mechanisms that closely link rewards to performance. Practically applied, this means that, e.g., a specific employee could be motivated to perform better if better job performance will lead to organizational rewards, such as an increase in salary. In this context, a crucial factor often missed is ensuring that the benefits provided are deserved and wanted by the recipients. While one individual could be motivated by salary another one could be motivated by, e.g., time off or specific training. Trainings for managers need to be organized by human resources to raise the knowledge about different motivational theories and strengthen leadership qualities. Managers who have internalized the theory can therefore provide an answer to the question of what determines their individual employees’ motivation by evaluating individual employee’s personality, skills, and knowledge without generalizing their veterinary staff and through this increase motivation and health of the individual employee. At the same time, employees whose basic needs are met and who are aware of this theory can more easily evaluate which benefits motivate them and communicate this to management.

Based on the already available studies investigating equity theory in human health care settings, veterinary managers applying this theory could reduce sector-specific problems like burnout and suicide rates and improve the quality of life for employees. Burnout is strongly related to turnover and reductions, which exacerbates staffing shortages and leads to a dangerous cycle that further increases the workload of the remaining staff. This is because Adams equity concept has far-reaching ramifications for staff morale, productivity, efficiency, and turnover. Human resources specialists and veterinary managers realizing or even preventing an employee from experiencing inequity could influence these sector-specific problems through intervention. Employees themselves would certainly find it easier to communicate openly when they experience inequality once they know the concept of inequity, as this knowledge certainly helps to sort out negative experiences and analyze the reason one experiences them.

Another method that can be used by veterinary managers or human resources is the goal-setting method. If managing staff would set specific, measurable, attainable, and time-related goals in collaboration with the employee, their motivation would be optimized. Giving constructive feedback time and space is something that still must be learned by most veterinary organizations. This allows the individual to improve. Feedback can be given mutually: both from the employee to the management and from the management to the employees. In this way, constructive dialog can be stimulated.

Optimal communication, cooperation, and feedback discussions within the team could lead to an increase in employee motivation by utilizing the introduction of the goal theory. Still, mainly McClelland’s theory, as well as the attribution theory, reflect the importance of the application of general psychology in the workplace.

### 4.2. Limitations

When interpreting the findings of this scoping review, several limitations should be considered. The review excluded unpublished or grey literature. In addition, articles that were not published in English or German were excluded, probably resulting in a loss of global information resulting in an enormous undertaking for this scoping review. Many papers included focused motivation, the healthcare profession, nurses, motivation in work psychology, and motivation theories. Most existing research data focus on the healthcare sector, which benefits from the existing theories. More research is needed for the veterinary healthcare sector, which can be done in further research using meta-analysis and qualitative approaches.

## Figures and Tables

**Table 1:** Inclusion criteria.

Inclusion Criteria	
Types of studies	English and German language only
	Worldwide studies
	Publication date 1930–2020
	Observational studies, mixed studies, empirical studies, review articles, and theoretical articles
	Peer reviewed journal papers Chapters with an abstract
Types of participants	Practicing and retired human and veterinary health care employees including students and junior trainees and specialists. Employees of other professions to compare the health care sector to other professions

**Table 2:** Exclusion criteria.

Exclusion Criteria	
Types of studies	Non-English/non-German language
	Proceedings
	Publication date before 1930 or after 2020
	Duplicates Editorials Letters Commentary Press release Grey literature
Types of participants	Regarding the articles investigating health care settings: non-practicing human and veterinary health care employees e.g., laboratory staff

## Supplementary Materials

The following supporting information can be downloaded at: https://www.mdpi.com/article/10.3390/bs13110898/s1, Table S1: Overview of reviewed articles.
