# Supplementary material for: When the Going Gets Challenging—Motivational Theories as a Driver for Workplace Health Promotion, Employees Well-Being and Quality of Life
Source: Behav Sci (Basel). 2023 Oct 30;13(11):898. doi: 10.3390/bs13110898 (PMC10669707; doi:10.3390/bs13110898)
Supplement: Supplementary file 1 [file behavsci-13-00898-s001.zip › behavsci-2564090-supplementary.pdf]

Table S1: Overview of reviewed articles

No	Reference	Title	Country	Motivational theory considered	Research approach	Population	Findings/Goal
1	Jodai et al (2013)	Motivation, Integrativeness, Organizational Influence, Anxiety, and English Achievement: Evidence from a Military University	Iran	Cognitive	Methodological using the mini-Attitude Motivation Test Battery	Iranian military staff (n= 164)	Finding: Motivation is positive factor in achievement of a goal
2	Kispál-Vitai (2016)	Comparative analysis of motivation theories. International Journal of Engineering and Management Sciences	Hungary	General psychology:<br>• Hedonism<br>• Instincts<br>• Drive reduction<br>• Arousal<br>• Incentive<br>• Cognitive<br>• Maslow's hierarchy of needs<br><br>Work psychology:<br>• Early<br>• Content<br>• Learning based<br>• Process	Theoretical<br><br>Comparative analysis	/	Finding: multidisciplinary research (general/work psychology/ clinical neuropsychology)creates new results crucial for practicing managers in designing effective motivational strategies for their employees
3	Steers et al (2004)	The future of work motivation theory	USA	Classical theories and adaptations of the 21st century	Review	/	Finding: Future of work motivation lies in adaptation of the classical theories to make them fit into todays world
4	Korenćić-Kampl (1996)	Survey about the motivation for the study of veterinary medicine in Croatia	Croatia	Factors affecting motivation	Methodological	First Year veterinary medicine students	Findings: candidates for the veterinary profession are motivated through direct affection to the profession and the love to animals

5	Brown (2007)	Psychology of motivation	USA	• Drive reduction<br>• Incentive<br>• Intrinsic/extrinsic<br>• Maslow´s hierarchy of needs	Theroretical	/	Goal: to assist the reader to understand and appreciate the complex issue of human motivated behaviors
6	Young (1936)	Psychological hedonism	USA	Hedonism	Theoretical	/	Goal: investigating hedonism as a motivator of behavior
7	McAllister (1953)	Toward a re-examination of psychological hedonism	USA	Hedonism	Theroretical	/	Finding: psyhocolgical hedonism still remains a topic for future studies
8	Garson (2016)	Two types of psychological hedonism	USA	Hedonism	Theoretical	/	Goal: distinguish two different forms of hedonism<br>Finding: a careful analysis of the different forms can provide a useful framework for the future
9	Williams (2018)	Psychological hedonism, hedonic motivation, and health behavior	USA	Hedonism	Theoretical	/	Goal: offering a new way of thinking about hedonism, updated based on recent theory an research in neuroscience and psychology
10	McDougall (1908)	The gregarious instinct	USA	Instincts	Theoretical	/	Finding: in a highly civilized society instincts are less important
11	Shrestha (2017)	Instinct Theory of Motivation	Japan	Instincts	Theoretical	/	Goal: brief overview of the theory and critical evaluation of it in todays world
12	Hull (1943)	Principles of behavior	USA	Drive reduction	Theoretical	/	Goal: explaining human behavior
13	Wolpe (1950)	Need-reduction, drive-reduction, and reinforcement: a neurophysiological view	South Africa	Drive reduction	Review	/	Goal: evaluation drive reduction from a neurophysiological view

14	Seward (1956)	Drive, incentive, and reinforcement.	USA	Drive reduction	Review	/	Goal: investigating drive reduction theory in reinforcement
15	Stennett (1957)	The relationship of performance level to level of arousal	Canada	Arousal	Methodological	Male students (n=31)	Finding: an inverted-U relationship exists between the level of arousal and performance leve
16	Reisenzein (1994)	Pleasure-arousal theory and the intensity of emotions	Germany	Arousal	Methodological	Psychology students (n=35 in part 1; n=56 in part 2)	Finding: arousal and the intensity of emotions needs further investigation and can not be generalized
17	Heckhausen (2013)	The anatomy of achievement motivation	Germany	Several	Theoretical	/	Goal: Overview of motivation
18	Shrestha (2017)	Arousal Theory of Motivation	Japan	Arousal	Theoretical	/	Goal: brief overview of the theory and the Yerkes-Dodson Law and critical evaluation of it in today's world
19	Tolman (1952)	A cognition motivation model	USA	Cognitive	Theoretical	/	Goal: development of a cognitive motivation model
20	Piaget (1964)	Cognitive development in children	Switzerland	Cognitive	Theoretical	/	Goal: overview of cognitive development
21	Dermer (1975)	The interrelationship of intrinsic and extrinsic motivation.	USA	Cognitive	Methodological	Store managers (n=81)	Goal: investigating interrelationship between intrinsic/extrinsic motivation
22	Pinder (2014)	Work motivation in organizational behavior	USA	Several	Theoretical	/	Goal: overview of development of work motivation theories, concepts of work motivation and determinants of behavior

23	Kuvaas et al (2017)	Do intrinsic and extrinsic motivation relate differently to employee outcomes?	Norway	Cognitive	Methodological	Employees (n=557) and store managers (n=106) of gas stations	Findings: intrinsic motivation is associated with positive outcomes. Extrinsic motivation is negatively related/unrelated to positive outcomes. Intrinsic/extrinsic motivation are moderately negatively correlated

No	Reference	Title	Country	Motivational theory considered	Research approach	Population	Findings/Goal
24	Turner (2017)	How does intrinsic and extrinsic motivation drive performance culture in organizations?	USA	Cognitive	Review		Findings: there is a mixed approach in whether employee's motivation is intrinsic or extrinsic. The type of motivation is also based on one's role within their organization.
25	Legault (2016)	Intrinsic and extrinsic motivation	USA	Cognitive	Theoretical	/	Goal: Overview of intrinsic/extrinsic motivation in today's world
26	Mullins (2007)	Management and organisational behavior	UK	Several	Theoretical	/	Goal: overview of management and organizational behavior in today's world
27	Dinibutun (2012)	Work motivation: Theoretical framework	Turky	Content theories:<br>• Maslow,<br>• Alderfer,<br>• McClelland<br>• Herzberg<br>Process theories:<br>• equity theory<br>• expectancy theory	Theoretical	/	Goal: exploring different theories of motivation, and presenting motivation as a basic psychological process
28	Maslow (1954)	Motivation and Personality	USA	Maslow´s hierarchy of needs	Theoretical	/	Goal: introduction of a new theory

29	Ball (2012)	A summary of motivation theories	Germany	• Maslow's hierarchy of need<br>• Alderfer's ERG<br>• McClelland'sAchievement<br>• Adam's Equity<br>• Herzberg's two factor<br>• Vroom's expectancy<br>• Hackman&Oldham's Job Characteristics Model	Theoretical	/	Goal: overview of the motivation theories and their use in today's world
30	Mangi et al (2015)	Motivation tools and organizational success: A critical analysis of motivational theories	Pakistan	• Maslow's hierarchy of needs<br>• Herzberg's 2 factor theory<br>• Vroom's expectancy theory<br>• Alderfer's ERG theory	Review	/	Goal: Dynamic view of motivation and its impact on the organizational achievement. Discussion of major issues and emphases of recent work that opens new basis to further research
31	Alderfer (1972)	Existence, relatedness, and growth: Human needs in organizational settings	USA	Alderfer's ERG theory	Theoretical	/	Goal: introduction to the ERG theory

32	Stello (2011)	Herzberg's two-factor theory of job satisfaction: An integrative literature review	USA	Herzberg's 2 factory theory	Review	/	Finding: it stands the time, integrates itself into basic points of view about management, and continues to provide ideas for new generations of scholars. It has proven its value.
33	Alshmemri et al (2017)	Herzberg's two-factor theory	Australia	Herzberg's 2 factory theory	Review	/	Findings: The results support Herzberg's theory and assert that the hygiene factors are less important to job satisfaction; the motivation factors are most important and can lead to job satisfaction. The theory identifies and explains the phenomena of job satisfaction.
34	Herzberg (1966)	Work and the Nature of Man	USA	Herzberg's 2 factory theory	Theoretical	/	Goal: Introduction to the 2-factor theory
35	McClelland (1961)	Achieving society	USA	achievement motivation theory	Theoretical	/	Goal: Introduction to the achievement motivation theory

36	Pardee (1990)	Motivation Theories of Maslow, Herzberg, McGregor & McClelland. A Literature Review of Selected Theories Dealing with Job Satisfaction and Motivation	USA	• Maslow,<br>• Herzberg,<br>• McGregor<br>• McClelland	Review	/	Findings: Managers incorporating motivational elements in work settings rather than simply eliminating adverse hygiene factors, they can expect higher productivity and job satisfaction.
37	Vroom (1964)	Work and motivation	Canada	Expectancy theorie	Theoretical	/	Goal: Introduction to the expectancy theory
38	Porter and Lawler (1968)	Managerial attitudes and performance	USA	Expectancy theorie	Theoretical	/	Goal: expanding the expectancy theory
39	Adams (1963)	Towards an understanding of inequity	USA	Equity theory	Theoretical	/	Goal: Introduction to the equity theory
40	Locke (1968	Toward a theory of task motivation and incentives	USA	Goal setting theory	Theoretical	/	Goal: introduction to the goal setting theory
41	Heider (1958)	The Psychology of Interpersonal Relations	USA	Attribution theory	Theoretical	/	Goal: introduction to the attribution theory
42	Kelley (1973)	The processes of causal attribution	USA	Attribution theory	Theoretical	/	Goal: introduction to the attribution theory

43	Ganta (2014)	Motivation in the workplace to improve the employee performance	India	Cognitive<br>Maslow´s theory<br>Herzberg's theory	Review	/	Goal: to emphases the importance of motivation in the workplace/to improve the employee performance and productivity/to present the theories and techniques of the motivation in the workplace
44	Yerkes and Dodson (1908)	The relation of strength of stimulus to rapidity of habit-formation	Canada	Yerke-Dodson law<br>Arousal theory	Methodological	Mice (n=40)	Goal: experiments and introduction to the Yerke-Dodson law
45	Ryan (2000)	Self-determination theory and the facilitation of intrinsic motivation, social development, and well-being	USA	Cognitive	Theoretical	/	Findings: intrinsic motivation supports psychological well-being more than extrinsic motivation
46	Karasek (1979)	Job demands, job decision latitude, and mental strain: Implications for job redesign	USA	General	Methodological	National surveys from Sweden and the USA evaluation stress models	Goal: redesigning work processes to allow increases in decision latitude for a broad range of workers to reduce mental strain and do so without affecting the job demands that may plausibly be as- sociated with organizational output levels.

47	Meyer (1997)	Commitment in the workplace: Theory, research, and application	UK	General	Review	/	Goal: Give an overview over available research regarding commitment to the workplace
48	Mackay (1987)	Occupational stress and health. International review of industrial and organization psychology	UK	General	Review	/	Goal: Give an overview on international research grading steps and health in the workplace
49	Lazarus (1995)	Psychological stress in the workplace	USA	General	Theoretical	/	Goal: describing the current theoretical perspectives and empirical work in occupational stress
50	Driscoll (2022)	Credentialed veterinary technician intrinsic and extrinsic rewards: a narrative review	USA	Cognitive	Review	/	Goal: provide an economic overview of the veterinary technician profession based on intrinsic and extrinsic rewards<br>Finding: veterinary technician intrinsic and extrinsic rewards are complex; changes to the profession, such as increasing veterinary technician utilization, should simultaneously incorporate the economic needs and values of veterinary technicians and veterinary practice operators.

51	Wynia (2009)	The risks of rewards in health care: How pay-for-performance could threaten, or bolster, medical professionalism	USA	General	Theoretical	/	Finding: profession has to asset its responsibility for determining how to define, measure and reward for quality—then pay-for-performance can promote not only high-quality care, but other core professional values too, including professional autonomy, collegial oversight and self-regulation.
52	Iglehart (2013)	Expanding the role of advanced nurse practitioners--risks and rewards	USA	Cognitive	Theoretical	/	Goal: discussing possibilities to address the nursing shortage in the US
53	Morgan et al (2013)	The quality of healthcare jobs: can intrinsic rewards compensate for low extrinsic rewards?	USA	Cognitive	Methodological	Front line workers in 25 health care organizations in the USA (n= 1006)	Goal: examines the influence of extrinsic job characteristics (e.g. wages and benefits) versus intrinsic characteristics (e.g. meaningful tasks) on job satisfaction and intent to stay with one's current employer Finding: both intrinsic and extrinsic characteristics are significant predictors of job satisfaction, but only extrinsic characteristics help explain intent to stay with the employer.

54	Ganster at al (2013)	Performance-based rewards and work stress.	UK	Cognitive	Review	/	Goal: linking performance-based reward systems to various indicators of employee stress and well-being.<br>Findings: reward systems generally deserve much more attention in the work stress literature
55	Victor and Hoole (2017)	The influence of organisational rewards on workplace trust and work engagement	South Africa	Cognitive	Methodological	South African employees in various industries within the Gauteng region (n= 251)	Goal: to determine the relationship between rewards, trust and engagement, and to investigate if rewards are able to predict trust and engagement<br>Findings: moderate-to-strong positive relationship between the three constructs, and that rewards are able to predict trust and engagement.
56	Triffin (1979)	Industrial Psychology	USA	General	Theoretical	/	Goal: overview of organizational and social context of human work

57	Kacel et al (2005)	Measurement of nurse practitioner job satisfaction in a Midwestern state	USA	General	Methodological	Randomized sample of licensed nurses (n= 147)	Goal: To describe the current level of job satisfaction of nurse practitioners<br>Findings: Population was minimally satisfied to satisfied: most satisfied with intrinsic factors and least satisfied with extrinsic factors of the job
58	Lephalala (2006)	Factors influencing nursing turnover in selected private hospitals in England	South Africa (author)/UK (study)	Herzberg's 2 factory theory<br>Cognitive	Methodological	Nurses in selected private hospitals in England (n= 85)	Findings: turnover rates might be reduced if promotion policies could be consistent, doctors would value nurses' work and if nurses could be more autonomous. Enhanced communication about policies and about accessing information and improved salaries could contribute to reduced turnover rates

59	Mitchell (2009)	Job satisfaction and burnout among foreign-trained nurses in Saudi Arabia	Saudi Arabia (study)/USA (author)	Herzberg's 2 factory theory	Methodological	Saudi Arabian nurses with international training (n= 453)	Findings: motivators work recognition, salary, working conditions, accomplishment, corporate policy and administration, and connections with managers and coworkers; employment discontentment: business policy and administration, working circumstances, status, supervisor relationships, security, and personal life
60	Osemeke and Adegboyega (2017)	Critical review and comparism between Maslow, Herzberg and McClellands theory of needs	Nigeria	Maslow, Herzberg, McClelland	Review	/	Findings: organizations should endeavor to adopt McClelland's theory to motivate their employees
61	Slocum and Hellriegel (2009)	Principles of organizational behavior	USA	Several	Theoretical	/	Goal: provide a holistic understanding of management in today's business world.
62	Van Dierendonck et al (1994)	Burnout among general practitioners: a perspective from equity theory	The Netherlands	Equity theory	Methodological	General practitioners (n= 567)	Findings: equity theory enhances understanding of the relationship between practitioner and patient as a determinant of burnout

63	Habibipour (200)	The effect of implementing “Goal Setting Theory” by nurse managers on staff nurses’ job motivation	Iran	Goal setting theory	Methodological	Nurses from one Nigerian hospital (n= 30 experimental population; n= 21 control population)	Findings: Implementing the “goal setting theory” resulted in a higher level of job motivation in staff nurse in this study
64	Weiner (2004)	Social motivation and moral emotions'. Attribution theory in the organizational sciences	USA	Attribution theory	Theoretical	/	Goal: Overview of attribution theory in todays organizational world
65	Weiner (2008)	Reflections on the history of attribution theory and research: People, personalities, publications, problem	USA	Attribution theory	Theoretical	/	Goal: Overview of attribution theory in todays organizational world
